# Everybody's Page

**Published:** 1892-12-03

**Authors:** 


### EVERYBODY'S PAGE.

Among many good answers to examination questions the following may fairly be included. A fair student, who was being examined in the Acts of the Apostles, defined "Euroclydon" as "A wind which blows from all quarters at once—hence its danger."

The value of tea as a restorative agent has been pointed out in these columns. Its superiority over brandy in many cases is beyond question. As a writer in the "Quarterly" points out, the idea still lingers that alcohol keeps out the cold. As a matter of fact, mountaineers have found by repeated experience not only that the opposite of this holds true, but that alcohol is equally valueless as a cure for giddiness.

Those who look upon the Lord Mayor's show as a nuisance and an anachronism are usually voted very superior people. That these shows, managed as they are, are not only useless but can be harmful, will be admitted by most intelligent people. How senseless it is to take away from their spheres of action bodies of public servants who exist for the purpose of protecting lives and property was painfully illustrated by the fire at Luton which resulted fatally to one man, owing to the absence in London of the greater part of the fire brigade. The desire of the multitude to see a pagent is a perfect harmless and understandable one, which even superior people would hardly object to being gratified, if only the pageant were governed by reason and guided by sense. The Corporation could always find plenty of unemployed, who would be delighted to take part in the procession for a small consideration, and who could be disguised as firemen, mandarins, or anything else likely to commend istelf to a not hyper-critical multitude.

The great obstacle to the practically universal adoption of eight o'clock closing in the East of London has been the opposition of one or two firms, which it is hoped will be overcome shortly. The meetings which have been held recently on the subject will, perhaps, help to secure this end.

The Adulteration Act is no exception to the rule that Acts of Parliament are invariably imperfect and seldom accomplish the end aimed at. The different way in which this Act is administered in different districts clearly shows how necessary it is to have matters defined more distinctly. This seems to be especially the case with margarine and chicory. Though coffee and chicory may be sold mixed provided the nature of the mixture is stated, there is nothing as the law at present stands to mark out clearly what proportions are legal.

The paper on the practical side of scientific temperance, read by Miss Anne W. Richardson at the autumnal conference of the British Women's Temperance Association, has now been issued in the form of a pamphlet. Those interested in the question, though they will find nothing new, will find the relation of alcohol to digestion, disease, and mortality concisely set forth. The alleged great increase of drinking amongst women of all classes has been brought prominently before the association lately. There were people long-sighted enough to raise their voices against the giving of licenses to grocers from the moment it was suggested to do so, but in vain. The folly of giving these licences has become only too patent now, and workers in the temperance cause look forward anxiously to the withdrawal of a temptation for which our legislature alone is responsible.

